# Development and evaluation of improved *in-house* procedure for radiolabelling of theranostic agent [^177^Lu]Lu-PSMA-617

**DOI:** 10.1186/s41181-026-00462-y

**Published:** 2026-06-13

**Authors:** Nino Jukić, Dražen Huić, Marijan Žuvić, Ivan Nemet, Sanda Rončević

**Affiliations:** 1 Department of Nuclear Medicine and Radiation Protection, University Hospital Centre Zagreb, Kispaticeva 12, Zagreb, Croatia; 2 Department of Chemistry, Faculty of Science, University of Zagreb, Horvatovac 102a, Zagreb, Croatia

**Keywords:** Radiopharmaceutical labelling, Quality control, TLC analysis, HPLC analysis, ICP-MS analysis, [^177^Lu]Lu-PSMA-617

## Abstract

**Background:**

Recent focus in the treatment of prostate cancer is directed towards the development and application of theranostic agents such as [^177^Lu]Lu-PSMA-617. It combines a PSMA-specific peptidomimetic with a therapeutical radionuclide ^177^Lu and it is used in radioligand therapy that selectively delivers ionizing radiation into tumor cells. This study reports the automated radiosynthesis and quality control of an *in-house* [^177^Lu]Lu-PSMA-617 preparation and a direct comparison of its properties with the reference product (Pluvicto^®^).

**Results:**

The radiopharmaceutical was obtained with > 95% radiochemical purity and > 96% radiochemical yield. The addition of ethanol improved radiochemical yield. The obtained product maintained > 98% radiochemical purity for up to 72 h in isotonic saline and human serum at 25 °C and 37 °C. The use of carrier-added ^177^Lu for the *in-house* product and non-carrier-added ^177^Lu for the reference product was confirmed by ICP-MS and gamma spectrometry methods.

**Conclusion:**

Comparative quality control demonstrated generally equivalent quality attributes of *in-house* prepared product and the reference product, supporting *in-house* product suitability for routine clinical application at activities up to 7.4 GBq. This experiment showed that *in-house* production of ^177^Lu-labeled theranostics is cost-effective method and can be automatized resulting in high quality and efficient production.

**Supplementary Information:**

The online version contains supplementary material available at 10.1186/s41181-026-00462-y.

## Background

Prostate cancer (PCa) is the most common cancer in elderly men with a significant number of PCa patients progressing to lethal metastatic castration-resistant prostate carcinoma (mCRPC) (Bray et al. 2018; Kirby et al. 2011; Watson et al. 2015). Prostate-specific membrane antigen (PSMA) is highly expressed in primary and metastatic lesions of prostate cancer (Smith-Jones et al. 2000). It is also known as glutamate carboxypeptidase type II transmembrane glycoprotein. This protein is also expressed in bone metastases and cancerous lymph nodes, but it is the most valuable molecular target for the diagnosis, staging and therapy of prostate carcinoma due to selective upregulation in 90–100% of prostate carcinoma lesions (Minner et al. 2011; Rybalov et al. 2014; Ananias et al. 2009; Silver et al. 1997).

Over the past decade various PSMA-binding tracers have been introduced into the clinical applications, mostly in the form of ligands labelled with gallium-68 or fluorine-18 (Fry et al. 2025; Sorensen et al. 2020). These radionuclides are crucial for imaging and locating tumorous lesions. In contrast, the most useful therapeutic radionuclide is ^177^Lu, beta-emitting isotope, which incorporation into a targeted ligand destroys malignant cells (Hunt et al. 2025). Lutetium-177 radionuclide with half-life of 6.67 days decays to 100% by emission of beta minus particles to the stable isotope hafnium-177 (^177^Hf) and it is suitable for transportation without significant loss of activity. Furthermore, the advantage of ^177^Lu or [^177^Lu]Lu-based ligands emerge from convenient and economic production which usually give high radiochemical purity and adequate specific activity. Low energy γ rays are emitted during the decay of ^177^Lu (112.9 keV and 208.4 keV) that enable theranostic intervention through simultaneous scintigraphy and dosimetry (Banerjee et al. 2015; Afshar-Oromieh et al. 2016; Seifert et al. 2020). The term “theranostic” combines both parts of the concept i.e., “*thera*” for therapy and “*nostic*” for diagnostic (Hennrich et al. 2022). Although not a theranostic pair per definition, two types of radiopharmaceuticals, [^68^Ga]Ga-PSMA-11 and [^177^Lu]Lu-PSMA-617, are commonly used in a radiopharmaceutical theranostic approach. The first agent is suitable for the PET imaging of prostate cancer and the latter for the radioligand therapy (RLT).

There are two routes for the production of ^177^Lu radioisotope. The direct route commonly starts with isotope ^176^Lu and in indirect route starting isotope is ^176^Yb. In direct route, which is referred as carrier added (ca.), isotope ^176^Lu is not completely removed in isotope separation process and usually leads to the side production of impurity from isotope ^177m^Lu (T_1/2_ = 160 days). In indirect route, which is referred as non-carrier added (n.c.a.), radionuclide ^177^Lu can be chemically isolated from ^176^Yb, which results with high specific activities and absence of impurity from ^177m^Lu (Hashimoto et al. 2003). Although the use of ca. isotope ^177^Lu for labelling purposes of radiopharmaceuticals has been approved by the FDA, the use of n.c.a. isotope ^177^Lu is preferred for the preparation of the *in-house* and commercially available Pluvicto^®^ agent (Vogel et al. 2021).

**Fig. 1:**
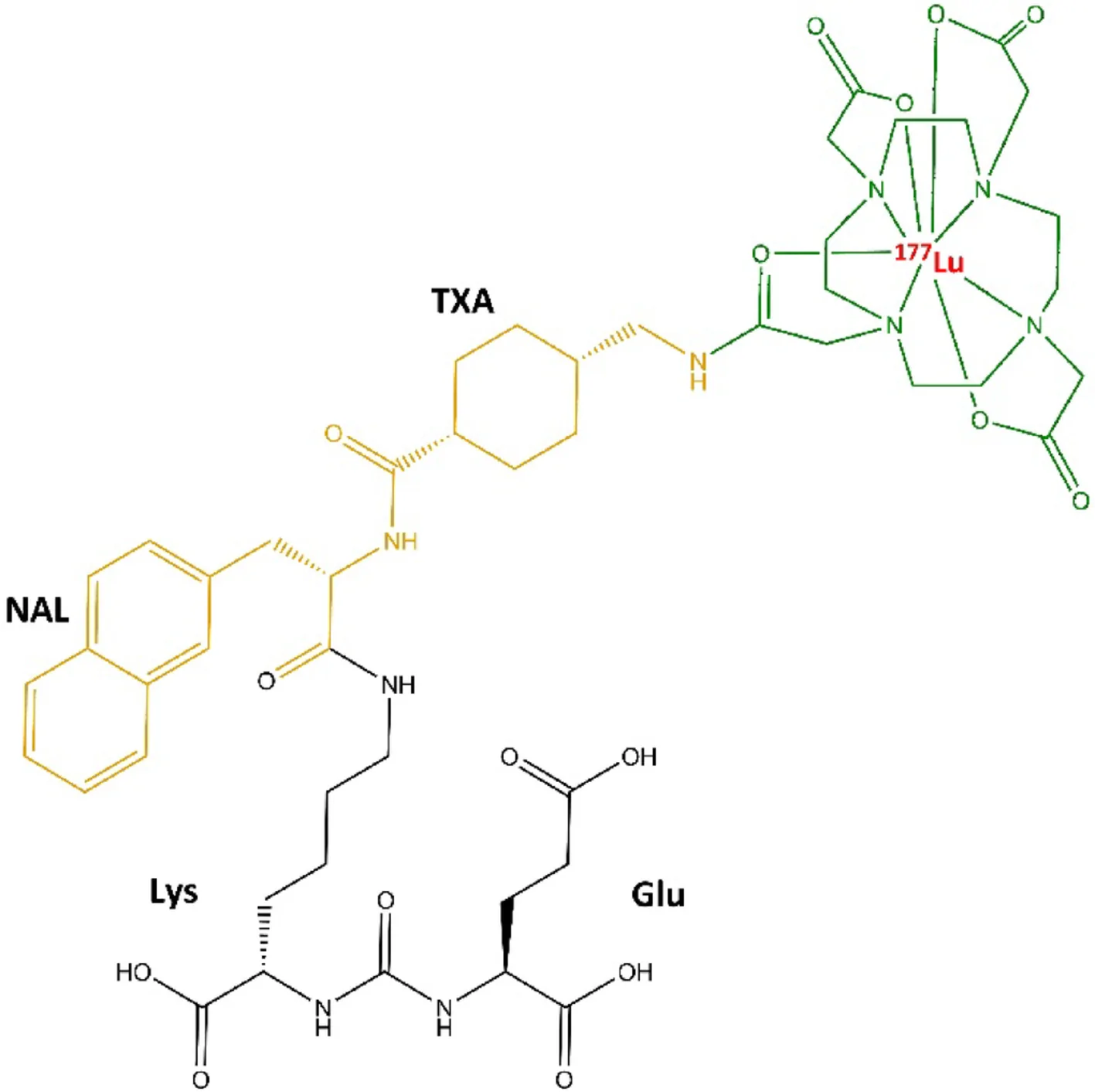
Chemical structure of [^177^Lu]Lu-PSMA-617 theranostic compound

Radiopharmaceutical [^177^Lu]Lu-PSMA-617, commercially known as Pluvicto^®^, is indicated for male adult patients who have prostate-specific membrane antigen (PSMA)-positive metastatic castration-resistant prostate cancer (mCRPC) and it was approved by FDA in March 2022 (Banerjee et al. 2015). The molecule of theranostic agent [^177^Lu]Lu-PSMA-617 consists of the PSMA-binding motif to which the DOTA (1,4,7,10-tetraazacyclododecane-1,4,7,10-tetraacetic acid) is coupled over a linker containing the amino acid 2-naphthyl-L-alanine (NAL) as well as tranexamic acid (TXA), which is shown in Fig. 1. This linker restores the high affinity to PSMA and high internalization into PSMA-positive LNCaP cells (Benešová et al. 2015). Octadentate coordination of DOTA allows stable coordination complexes with diverse radioisotopes (Aime et al. 1996). The complexes with isotopes ^68^Ga, ^225^Ac and ^177^Lu are mostly adopted for medical purposes.

Different approaches for the production of ready-to-use radiopharmaceutical [^177^Lu]Lu-PSMA-617 have been described in the literature. The radiolabelling with carrier-added (ca.) or no-carrier-added (n.c.a.) ^177^Lu with changing parameters such as ligand-to-metal ratio, reaction pH, reaction time, and product formulation. Furthermore, stabilization of the radiolabeled product with addition of radical scavengers such as DTPA, ethanol, ascorbic acid, or gentisic acid is often used to prevent radiolysis and maintain high radiochemical purity. (Peng et al. 2025; Martin et al. 2021; Chakraborty et al. 2016, 2018, 2021; Larenkov et al. 2024; Aalbersberg et al. 2022; Wichmann et al. 2021; De Zanger et al. 2019).

In this work, a procedure for the *in-house* radiochemical synthesis of the radiopharmaceutical [^177^Lu]Lu-PSMA-617 using automated module is described. The procedure for the formulation of therapeutic doses of [^177^Lu]Lu-PSMA-617 using ^177^Lu isotope of moderate specific activity (> 370 GBq mg^− 1^) along with variation of parameters such as ligand-to-metal ratio, pH value and addition of radical scavenger and complexing agent was examined. The quality of prepared products which is usually expressed through radiochemical yield and radiochemical purity is examined by use of HPLC and TLC methods. The aim of this paper is to present the advantages of the internal radioactive labelling procedure, as well as the results of quality control testing of the obtained product in comparison with the features of the reference product (Pluvicto^®^). Such comparisons are rare in the literature, and as far as we know, this paper presents for the first time a comparison of the quality of the reference and *in-house* prepared product. In order to characterize the properties of the prepared labelled compound before possible administration to patients, in vitro stability tests were also performed. Additionally, this work examines the possibility of ICP-MS and HPGe methods to determine which initial type of ^177^Lu isotope, carrier added or non-carrier added, is used in the preparation of [^177^Lu]Lu-PSMA-617 agent.

## Methods

### Chemicals and reagents

The solution of lutetium(III) chloride (^177^LuCl_3_); *A* = 4 GBq where radionuclide ^177^Lu is carrier-added (> 370 GBq mg^− 1^), and ascorbate buffer (*m* = 50 mg) were purchased from the Radioisotope Centre Polatom (Poland). Peptide PSMA-617 (vipovitide tetraxetan) was purchased from MedChemExpress (USA). Anhydrous LuCl_3_ (> 99.99% trace metal basis) and citric acid (anhydrous for synthesis) were purchased from Sigma-Aldrich (Milwaukee, WI USA). Pentetate zinc trisodium (Zn-DTPA) was purchased from Ditripentat Heyl^®^, Berlin, Germany. Ammonium acetate (for analysis), ethanol (Reag. Ph Eur.), methanol (hypergrade for LC-MS), acetonitrile (hypergrade for LC-MS), trifluoroacetic acid (for spectroscopy), and sodium acetate anhydrous (for analysis) were purchased from Merck (Germany). Water for injections (Aqua Pro) was purchased from the Croatian Institute for Transfusion Medicine (Zagreb, Croatia). Isotonic saline was purchased from Fresenius Kabi (Croatia). Hydrochloric acid (*c* = 0.04 mol L^− 1^) for dilution of ^177^LuCl_3_ solution was made from ultrapure HCl (*c* = 0.1 mol L^− 1^) which was purchased from Eckert&Zeigler (Germany). For ICP-MS analysis nitric acid (65% *v/v*) was purchased from Fischer Scientific (UK) further distilled on a Savillex DST-1000. Multi-element Calibration Standard 1 was purchased from Agilent Technologies (Santa Clara, CA USA). Syringe filter CHROMAFIL^®^, regenerated Cellulose (RC) was purchased from Macherey-Nagel GmbH & Co. KG (Germany). The pH of the buffer solutions, the ^177^LuCl_3_ solution, reference product and the products were determined using pH paper indicator strips (Supelco pH 2.0–9.0) purchased from Merck (Germany). For TLC, iTLC-SG chromatograph paper impregnated with silica gel (Agilent Technologies, Santa Clara, CA USA) was used. Limulus Amebocyte Lysate (LAL) cartridges for the determination of bacterial endotoxins were purchased from Charles River Laboratories (USA).

### Instrumentation

Thin-layer chromatography was performed by applying 5 µL of a solution on the iTLC-SG strips. The paper strips were eluted, dried, and scanned using a miniGita TLC scanner (Elysia Raytest, Germany). Multigamma Mixture reference source for HPGE detector was purchased from LEA (laboratoire etalnos d’activité, France). Activity measurements of ^177^LuCl_3_ solution, final product vials, reference product vial, and samples in insulin syringe for mesaurements on HPGe detector were done using a VIK-202/5051 dose calibrator (COMECER Netherlands). High-performance liquid chromatography (HPLC) analysis was performed on a Shimadzu Nexera XR equipped with an NaI(Tl) scintillation detector GabiStar Nova (Elysia Raytest, Germany) and a reversed-phase column (Luna C18, 150 × 4.6 mm, 5 microns, Phenomenex, USA). Mobile phase for HPLC experiments consisted of 0.1% TFA in water (solvent A) and 0.1% TFA in acetonitrile (solvent B). The flow rate was adjusted to 0.6 mL/min. Chromatograms were detected by UV-Vis detector (*λ* = 280 nm) after isocratic elution during 20 min with mixture of 22% acetonitrile and 78% H_2_O. Gas chromatography analysis was performed using a Shimadzu Nexis GC-2030 gas chromatograph equipped with a Shimadzu column SH-1, 30 m, 0, 53 mm ID. Nitrogen carrier gas was used. The flame ionization detector (FID) was set at 250 °C and oven temperature was programmed from 50 to 245 °C. Injection volume was 0,2 µL and time of analysis 10 min. Gamma spectrometry was performed on an HPGe detector, Canberra (USA), with software Gennie 2000. Energy and efficiency calibration of the detector were carried out using a Multigamma Mixture reference source prior to the recording of gamma ray spectra. Agilent 7900 ICP-MS was used with the following optimal operating conditions: radiofrequncy (RF) plasma power 1500 W, gas flow (Ar) 0,90 L min^− 1^, additional gas flow (Ar) 0,20 L min^− 1^, nebulizer *MicroMist*, nebulizer pump 0,10 rps, spray chamber *Scott double pass*, time of integration 0, 10 s, external calibration. Samples were analyzed in duplicate. Bacterial endotoxins in products and reference product were determined with Endosafe^®^ nexgen-PTS™ spectrophotometer (Charles River Laboratories, USA).

### Operating conditions and procedures for the radiolabelling by automatic module

Before placing the reagents on the cassette, the integrity of the cassette is visually checked. The strength of each joint on the cassette is checked so that each thread is secured by turning. At position B1, a sterile filter from needle is inserted. At position B2 there is a tube that is placed in the pressure sensor in the module. At position B3, the tube is connected to the water tank, which is hung on the hook on the right side of the module. An empty 5 mL injection syringe is placed in position B4.

It is usual to place a syringe containing the peptide with a buffer, but due to the small volume of the reagent during synthesis, the buffer and the peptide were added directly in the reaction vial after adding ^177^LuCl_3_ solution.

At position B5, a sterile filter with a Sterican needle 20G (0.90 × 70 mm) is connected to the product delivery tube. Left side of position B1 and right side of position B5 contains two tubes to which Sterican 20G needles are connected.

The module door opens. The cassette is placed on its side so that the valves are facing the person placing it. The central part with B positions is fixed with screws. A lead shield containing the product vial is placed on the right side of the module. The septum of the vial is wiped with a isopropanol (*w* = 70%) swab and the vial is vented with a needle with filter. In the next step, the product delivery tube with the filter and Sterican needle is placed in the product vial. The Sterican needle with filter is inserted to approximately 2/3 of the volume of the vial because the needle should remain above the liquid after the radiopharmaceutical delivery.

The tube that connects to the water tank is threaded through the slot on the module on the right side, connected to the water tank and hung on the hook on the right side of the module. On the left side of the module is the part of the device in which radiolabelling occures. It consists of an oven into which a reaction vial with reagents is placed. Two tubes are threaded through the module slot on the left side: one that continues to the left from position B1, and one that continues to the right from position B5.

The tube that continues to the left is simply threaded through the module slot to the reaction vial. The tube that continues to the right is pressed into the module pump, closed with a flap and threaded left through the slot of the module to reaction vial. Both tubes contain Sterican needles, the upper plastic part of of the needle is inserted into the plastic slots of the module. The needles are set in the V-position and secured with a lever. The tube at position B2 is connected to the pressure sensor of the module.

Radiopharmaceutical [^177^Lu]Lu-PSMA-617 was synthetized by the Gaia&Luna module (Elysia Raytest, Germany). Radiochemical labelling was performed using a fluidic kit for ^177^Lu-labelling of peptides together with a reagent kit (ABX, Germany). The reagent kit contains the following components: a waste vial, a mixing vial, a female Luer lock coupler, water for injections (Ph. Eur.), a 25 mL evacuated product vial, a 0.2 μm syringe filter (Millex GV), a sterile 5 mL syringe and a three sterile Sterican needles. All synthesis occurred in a reaction vial (*V* = 10 mL) with a flat bottom sealed with a bimetal magnetic crimp cap. The ^177^LuCl_3_ solution was first added to the reaction vial, followed by the buffer and peptide.

**Fig. 2:**
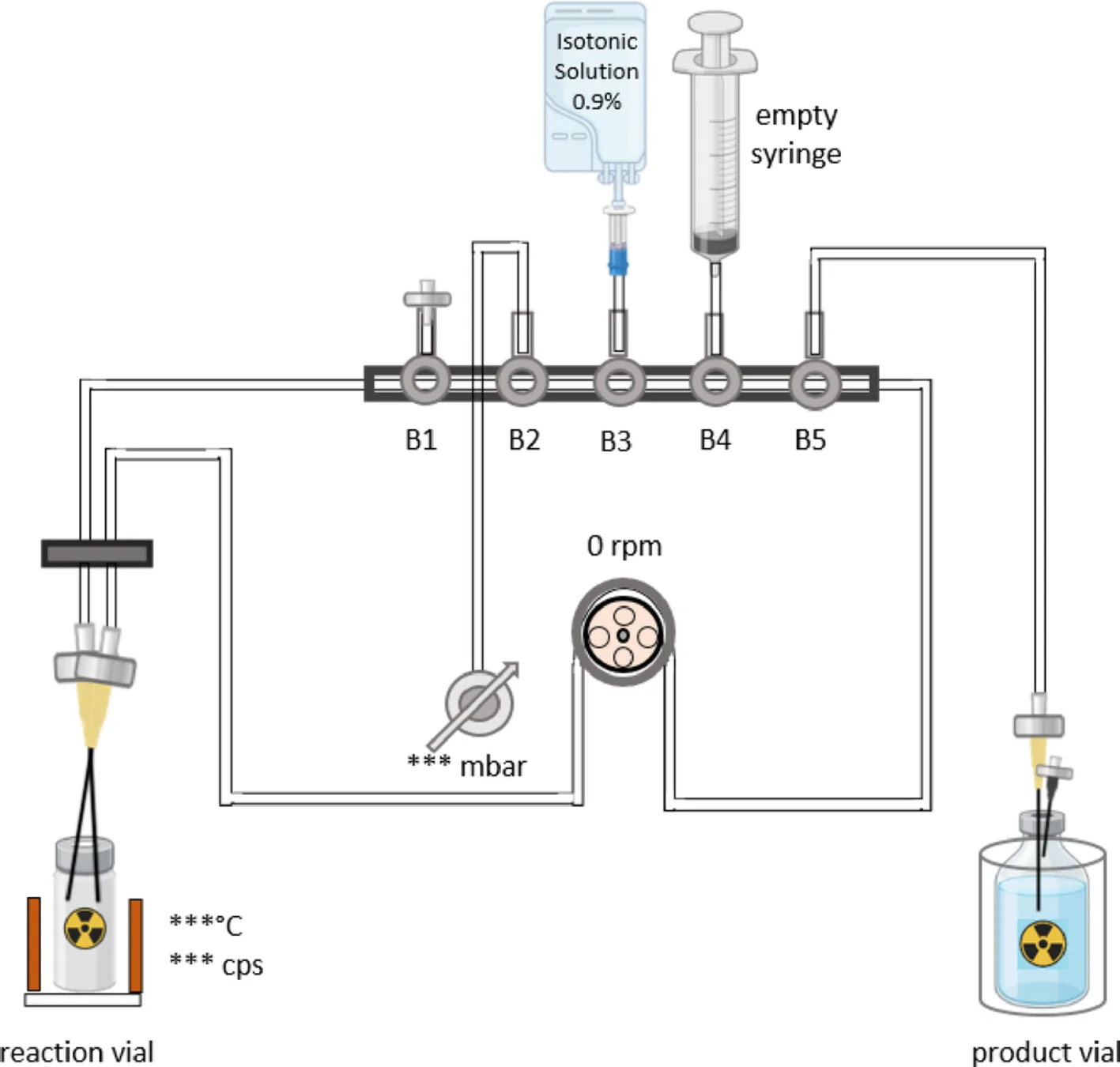
Schematic diagram of automatic module for the radiolabelling of ^177^Lu radiopharmaceuticals

The preparation of the radiopharmaceutical [^177^Lu]Lu-PSMA-617 was performed using the automated module and lasts 30 min at 90 °C and consists of the following steps: (1) lowering the elevator with yellow needles through the septum of the reaction vial; (2) cassette integrity test, where the cassette is subjected to a pressure of 2500 mbar, which checks the permeability of the cassette; (3) lifting the elevator with yellow needles from the reaction vial; (4) labelling the peptide with radionuclide ^177^Lu for 30 min at 90 °C; (5) cooling the heater to 40 °C and turning it off; (6) lowering the elevator with yellow needles through the septum of the reaction vial and diluting the product with saline three times, and rinsing the system between dilutions; (7) moving the product needle to the waste vial manually; (8) end of synthesis. In Fig. 2 there is a schematic representation of the cassette with reagents for the synthesis of ^177^Lu radiopharmaceuticals. Filter integrity test was performed after radiolabelling for every batch as a separate method.

### Variation of parameters in radiochemical labelling of [^177^Lu]Lu-PSMA-617

In order to achieve the highest possible radiochemical purity and yield, a total of 9 labelling modes were tested during the experiment by changing the pH value and ligand to metal [L] / [M] ratio, where [L] is the amount of PSMA-617 peptide and [M] is the amount of lutetium (III) chloride (^177^LuCl_3_) solution and the addition of ethanol and Zn-DTPA as radical scavenger and complexing agent. The synthetical routes were performed using three approaches which are summarized in Table 1.

**Table 1:** Radiolabelling conditions and parameters used for the preparation of [^177^Lu]Lu-PSMA-617

Batch	A(^177^LuCl_3_) / MBq	V(^177^LuCl_3_) / µL	m(PSMA-617) / µg	Variation parameters		
				EtOH/µL	Zn-DTPA/µL	pH
1	195.3	85	20	–	–	4.5
2	211.6	110	20	–	–	5.0
3	236.2	160	20	–	–	5.5
4	237.1	180	20	20	–	5.5
5	658.4	290	20	–	–	5.5
6	127.1	90	20	–	–	5.5
7	237.4	250	20	–	83.8	5.5
8	236.7	145	20	–	1	5.5
9	172.5	125	20	27.2	–	5.5

In batches 1, 2, and 3 the pH value of the ascorbic acid buffer was adjusted to pH = 4.5, 5.0, and 5.5, respectively (*c* = 0.28, 0.19 and 0.16 mol L^− 1^). The amount of the peptide (20 µg) and the activity of the ^177^LuCl_3_ solution retained at the same levels (200 MBq) by varying added volume of solution in the first three batches. A solution of ^177^LuCl_3_ (I) (*A* = 2 GBq) in a volume of 0.013 mL was used. To obtain a specific activity of 2 GBq mL^− 1^, the solution was further diluted to 1 mL using 0.04 mol L^− 1^ of ultrapure HCl.

In the second mode of the experiments, the addition of radical scavengers was also tested. Ethanol was added in batches 4 and 9. In batch 4, which was adjusted to pH = 5.5, ethanol (50% *m/v*) was added, which made up 7% of the total reagent volume and in batch 9, ethanol (50% *m/v*) made up 10% of the total reagent volume.

Addition of Zn-DTPA was implemented in batches 7 and 8. In all preparations, Zn-DTPA solution was diluted to 2.11 mg mL^− 1^. The Zn-DTPA radical scavenger was added at 20 molar excess over PSMA-617 into the reaction vial immediately at the beginning of labelling (in batch 7). In batch 8, Zn-DTPA was added at the end of the preparation with an incubation period of 15 min. Here, it was added in quantity which was estimated from the concentration of free ^177^Lu^3+^ ions in product. In batches 5 and 9 ligand to metal [L] / [M] ratio was changed. It was ~ 2 in batch 5 and ~ 10 in batch 9, while in all other batches it ranged between 5 and 7. In the second approach, the specific activity of 2 GBq mL^− 1^ in ^177^LuCl_3_ solution was retained for batches 5–9.

### Evaluation of the quality of the product [^177^Lu]Lu-PSMA-617

Radiochemical purity of the *in-house* prepared products and the reference product (Pluvicto^®^, R) and radiochemical identity of the ^nat177^Lu-PSMA-617 were determined with HPLC method (Decristoforo 2018). Inactive reference compound ^nat177^Lu-PSMA-617 was prepared using *in-house* procedure. This product was filtered through a Millex filter in a sterile tube. (Kraihammer et al. 2023). Calibration curve was constructed by measuring four standard solutions of PSMA-617 peptide which were prepared in the concentration range of 4–50 µg mL^− 1^. Standard solutions were injected three times and the calibration curve was constructed from the areas of the peptide peak detected by UV detector. All HPLC analyses were performed by injecting 10 µL of the solutions in an isocratic mode of operation. Ethanol content in two products was determined by a Shimadzu Nexis GC-2030 gas chromatograph. The percentage of the ^177^Lu in colloidal form and free ^177^Lu^3+^ ions in products and reference product was determined using a TLC scanner with iTLC-SG strips. Solution of methanol and ammonium acetate (*c* = 0.1 mol L^− 1^) in ratio 1:1 *v/v* was used as mobile phase for determination of ^177^Lu colloid. Solution of sodium citrate dihydrate (*c* = 0,1 mol L^− 1^, pH = 5) was used for the determination of free ^177^Lu^3+^ ions. The radionuclidic purity of *in-house* prepared [^177^Lu]Lu-PSMA-617, the reference product and the ^177^LuCl_3_ solution was determined after 7 days of preparation by using HPGe detector. For this test, a volume of 1000 mL of water is added in Marinelli beakers which was followed by addition of amount that corresponds to activity of 500 kBq of prepared product, which was added in each vessel and measured lasting 2 h. Three regions of interest, i.e., energies that are characteristic for radionuclide ^177^Lu (*E*_*γ*_ = 113, 208, and 228 keV) were monitored.

### Stability assay in vitro

Stability of all prepared [^177^Lu]Lu-PSMA-617 products and reference product was studied in vitro using isotonic saline and human serum samples. Bacterial endotoxins were tested in all prepared products and reference product before performing in vitro tests. For this purpose, spectrophotometric method was used (automated system Endosafe PTS Nexgen). Human serum for stability studies in vitro was obtained from healthy workers at the Clinical Department of Nuclear Medicine and Radiation Protection at University Hospital Centre Zagreb. An aliquot of 200 µL of the *in-house* prepared radiopharmaceutical was mixed with 200 µL of isotonic saline or serum in sterile tubes and placed in a microbiological incubator at 25 °C and 37 °C during the incubation period of 72 h. The percentage of the *in-house* prepared complex [^177^Lu]Lu-PSMA-617 and the reference product was determined after 24 h, 48 h and 72 h using TLC scanner. Serum samples were injected directly and analyzed by HPLC and the stability of the radiopharmaceutical was estimated by the changes in the peak profile and retention time in the HPLC radiochromatogram after 24 h, 48 h and 72 h. A total of 96 prepared samples were analyzed using TLC and HPLC methods.

## Results

### Evaluation of purity by HPLC and TLC method

The procedure of *in-house* radiolabeling of the PSMA-617 peptide with radionuclide ^177^Lu using automatic module was examined by varying reaction parameters, such as the ligand to metal ratio, pH of the reaction mixture, and the addition of ethanol and Zn-DTPA as radical scavenger and complexing agent. Nine combinations of experiments of production of [^177^Lu]Lu-PSMA-617 were conducted and the final products were subdued to quality assessment procedures.

**Fig. 3:**
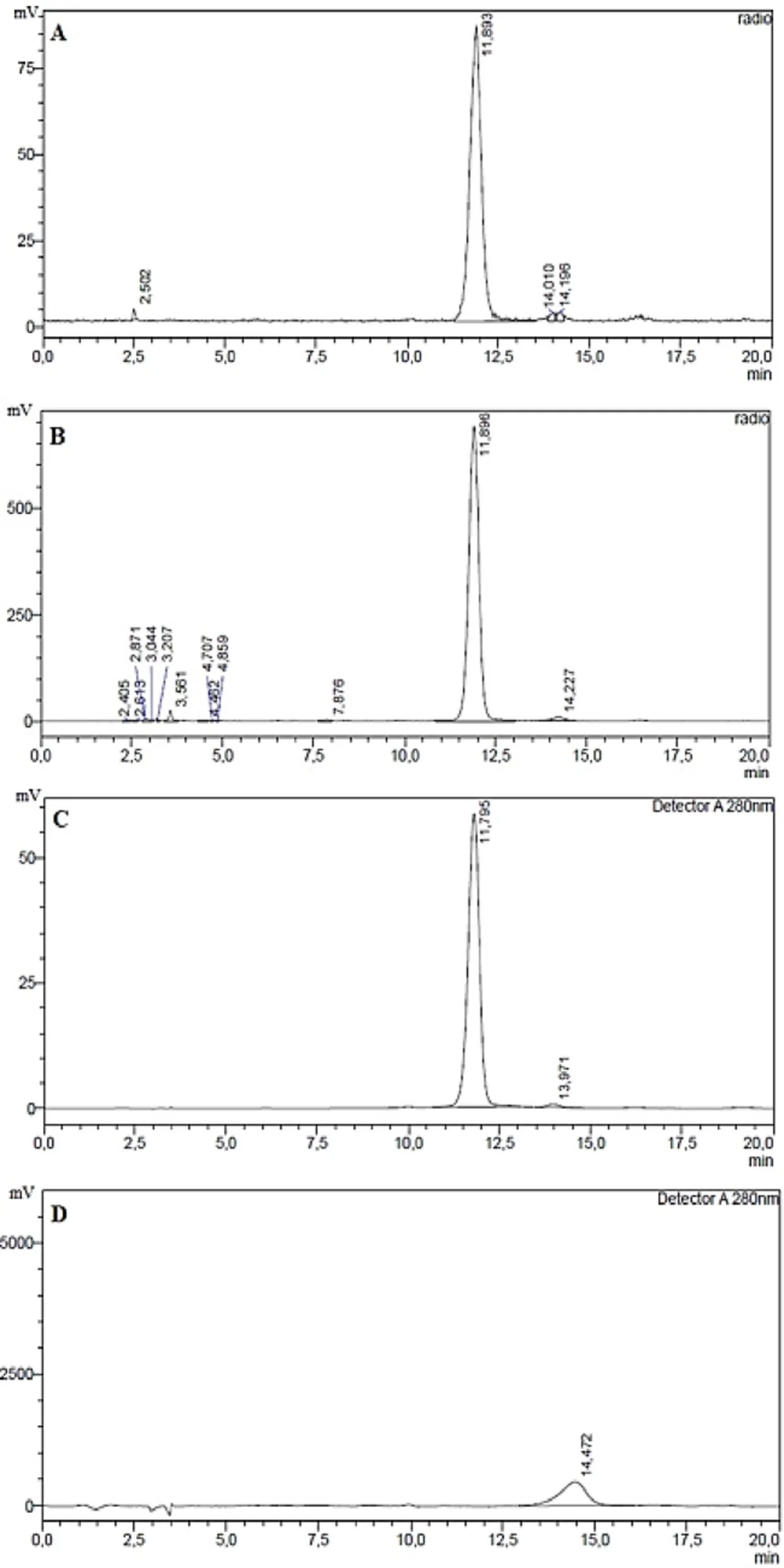
HPLC chromatograms of the radiosynthesized radiopharmaceutical [^177^Lu]Lu-PSMA-617 (**a**), reference product (**b**), inactive ^nat177^Lu-PSMA-617 (**c**) and peptide PSMA- 617 (**d**)

The radiochemical purity of obtained products was tested by HPLC method. Chromatogram of the *in-house* prepared radiopharmaceutical showed elution peak at retention time of 11.9 min (Fig. 3A). The peak denotes the free ^177^Lu^3+^ ions in injected sample solution appeared with retention time of 2.5 min. Reference product (Fig. 3B) showed the same retention time as the *in-house* prepared product. In this sample the retention time of free ^177^Lu^3+^ ions appeared at 2.4 min. In the UV chromatogram (Fig. 3C) of ^nat177^Lu-PSMA-617 and PSMA-617, the retention times were found at 11.8 min and at 14.0 min, respectively. In the UV chromatogram (Fig. 3D) of the peptide PSMA-617, the retention time was found at 14.4 min. Analysis of the 1:1 mixture of compounds ^nat177^Lu-PSMA-617 and PSMA-617 (the final concentration 50 µg/mL of each compound) provided a resolution of 4.22 that showed exceptional separation of PSMA-617 peptide from lutetium complex.

For the determination of radiochemical purity and radiochemical yield in all performed batches of labelling procedure, an HPLC instrument equipped with scintillation detector was used. In eight out of nine experiments, it was found that [^177^Lu]Lu-PSMA-617 product was formed with radiochemical yield greater than 95.0%, as showed in Table 2. Batch 3 showed the best radiochemical purity and the batches 4 and 9 showed the best radiochemical yield. It was observed that a complexation yield of 97% could be obtained using 20 µg (19.1 nmol) of PSMA-617 peptide at pH 4.5 of resultant mixture.

**Table 2:** Radiochemical yield and radiochemical purity of [^177^Lu]Lu-PSMA-617 products and reference product determined by HPLC method

Batch No.	Molar ratio [PSMA-617] / [Lu]	pH	RCY* (%)	RCP* (%)	Activity of ^177^Lu (EOS)*/MBq
1	6.36	4.5	91	97.3	178.0
2	5.87	5.0	94	96.1	199.5
3	5.26	4.5	94	99.1	223.2
4	5.24	4.5	97	95.7	229.4
5	1.89	4.5	96	96.0	631.7
6	9.77	4.5	83	96.8	106.1
7	5.23	4.5	99	0.63	235.4
8	5.25	4.5	93	97.2	221.1
9	7.20	4.5	97	96.7	167.0
R	-	5.5	-	95.6	10,313

^* EOS – end of synthesis; RCY – radiochemical yield; RCP – radiochemical purity^

HPLC and TLC methods were used in testing of impurities in products obtained in all batches by *in-house* radiochemical labelling (Table 3). Generally, the percentage of free ^177^Lu^3+^ ions was lower than 2% with the exception which is manifested in batch 7 where the radiolabelling was unsuccessful and all radionuclide content was eluted in free ion form.

**Table 3:** Radiochemical impurities from the free ^177^Lu^3+^ ions and colloidal form determined by HPLC and TLC methods

Batch No.	free ^177^Lu^3+^ (%) HPLC	free ^177^Lu^3+^ (%) TLC	^177^Lu-colloid (%) TLC
1	0.99	0.37	< LOQ
2	1.74	0.15	0.24
3	0.23	0.06	< LOQ
4	0.89	0.11	< LOQ
5	0.05	< LOQ	< LOQ
6	1.16	0.33	< LOQ
7	97.6	100.00	< LOQ
8	0.62	< LOQ	0.09
9	1.08	0.13	0.34
R	0.09	< LOQ	< LOQ

^*LOQ = 0.01%^

**Fig. 4:**
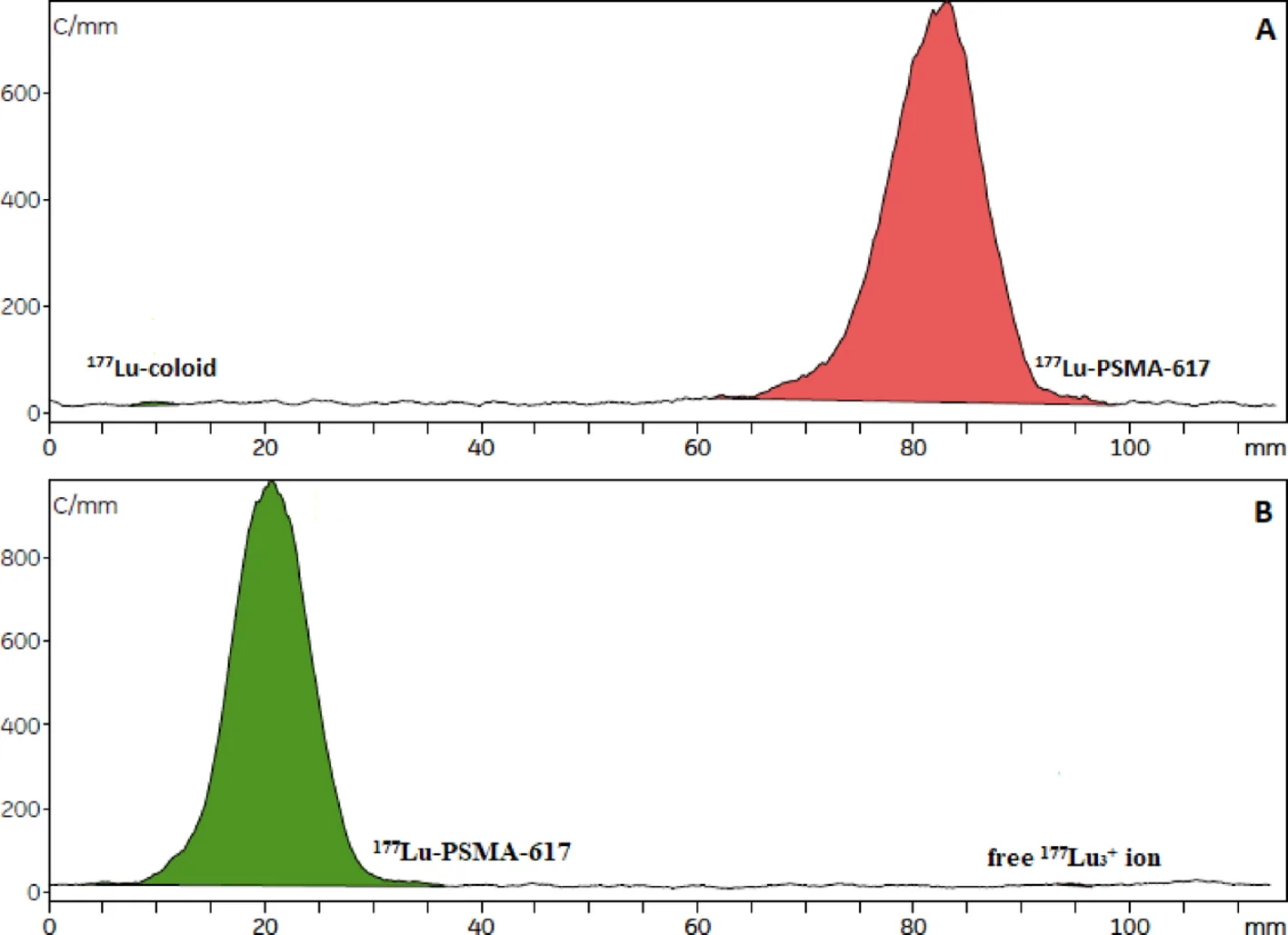
Thin-layered chromatograms (TLC) of the product 2 showing ^177^Lu-colloid remained at the spot of spotting (**a**) and non-complexed ^177^Lu^3+^ migrating to the solvent front (R_f_ = 0.9–1.0) (**b**)

The content of impurities from ^177^Lu colloidal form was determined only by TLC method. By using methanol and ammonium acetate (*c* = 0.1 mol dm^− 3^) in ratio 1:1 *v/v* solution with iTLC-SG, the ^177^Lu-colloid remained at the point of spotting (R_f_ = 0–0.3) while the non-complexed ^177^Lu^3+^ ions migrate to the solvent front (R_f_ = 0.9–1.0). An example of TLC chromatograms is showed in Fig. 4.

The amount of the peptide PSMA-617 after the radiolabelling procedure in all nine batches was determined by HPLC and results are presented in Table 4. In every batch an amount of 20 µg of the peptide was added at the beginning of radiolabelling procedure. Final measured results showed slightly bigger amount of the peptide in the products (20.8–31.4 µg) where mean value was 26.52 ± 3.14 (*n* = 9).

**Table 4:** Amount of the peptide PSMA-617 after the labelling procedures

Batch No.	Peak area/mV	m/µg	V_tot_/mL	γ _PSMA−617_/µg mL^− 1^	Effective specific activity (A_eff_)/ GBq µg^− 1^
1	770	20.8	6	3.4680	8.90
2	1363	24.1	6	4.0096	9.98
3	1166	26.8	7	3.8297	11.16
4	1275	31.4	8	3.9292	11.47
5	1083	26.3	7	3.7539	31.59
6	1506	29.0	7	4.1402	5.31
7	1549	29.3	7	4.1795	11.77
8	883	25.0	7	3.5712	11.06
9	1039	26.0	7	3.7137	8.35
R	4748	71.9	10.13	7.1009	143.44

### Radionuclidic stability

**Fig. 5:**
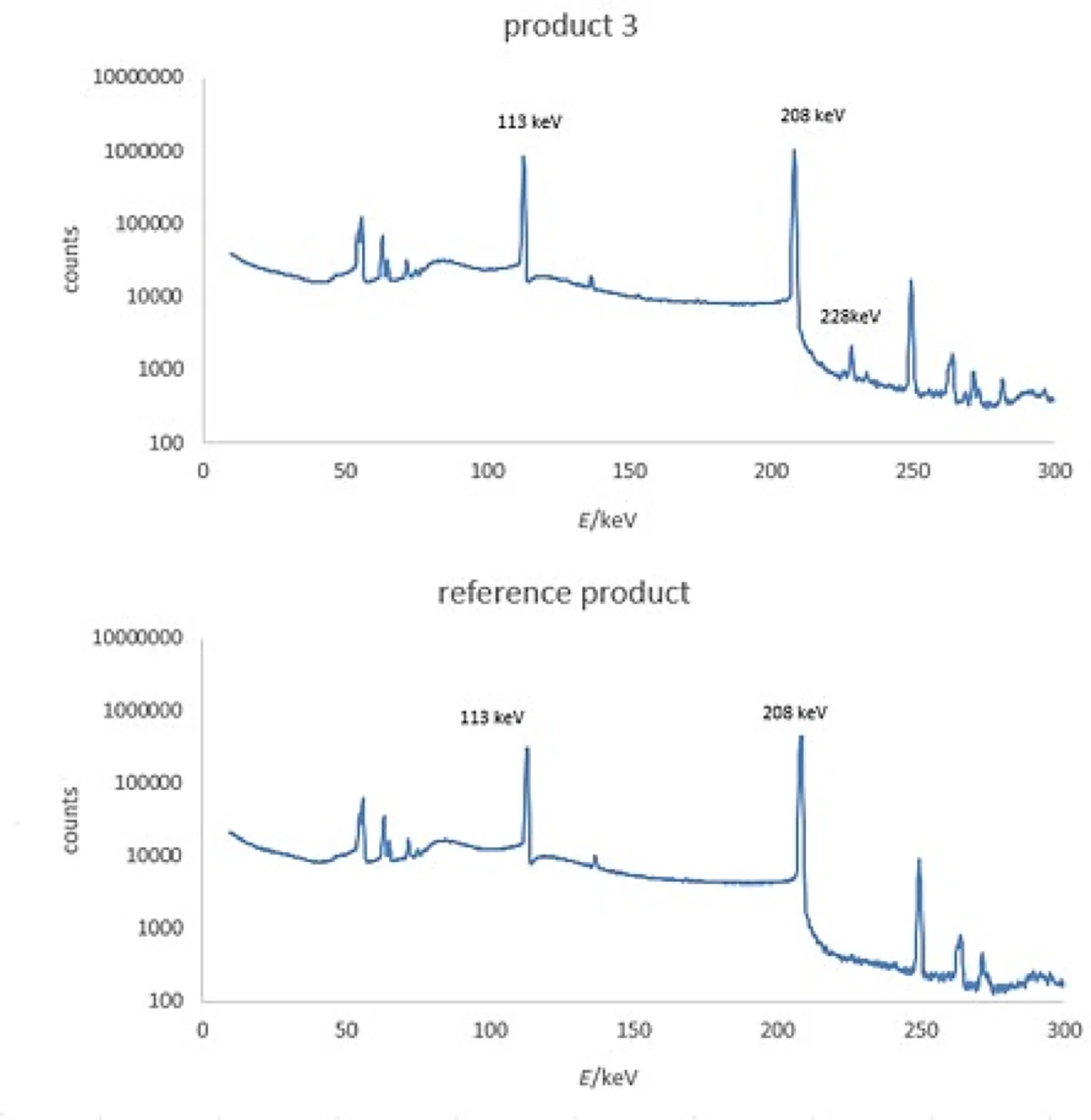
HPGe spectrum of the product 3 and the reference product showing characteristic gamma energies of the radionuclide ^177^Lu (*E*_*γ*_ = 113 and 208 keV) and the radionuclide ^177m^Lu (*E*_*γ*_ = 228 keV)

The radionuclidic stability of *in-house* prepared products, the reference product and ^177^LuCl_3_ solution was determined after 7 days by using HPGe detector. For the determination of the nuclide stability two energy peaks at 208 keV (^177^Lu) and at 228 keV (^177m^Lu) were considered. Energy peak at 113 keV (^177^Lu) was visible in the spectrum of the reference product and in all spectra of the *in-house* prepared products. For clarity, only the spectrum of batch 3 is presented, as it was obtained with highest radiochemical purity (Fig. 5). The peak of 228 keV was not visible in reference product spectrum, and this fact implies the mode of isotope production.

### Stability tests in vitro

**Fig. 6:**
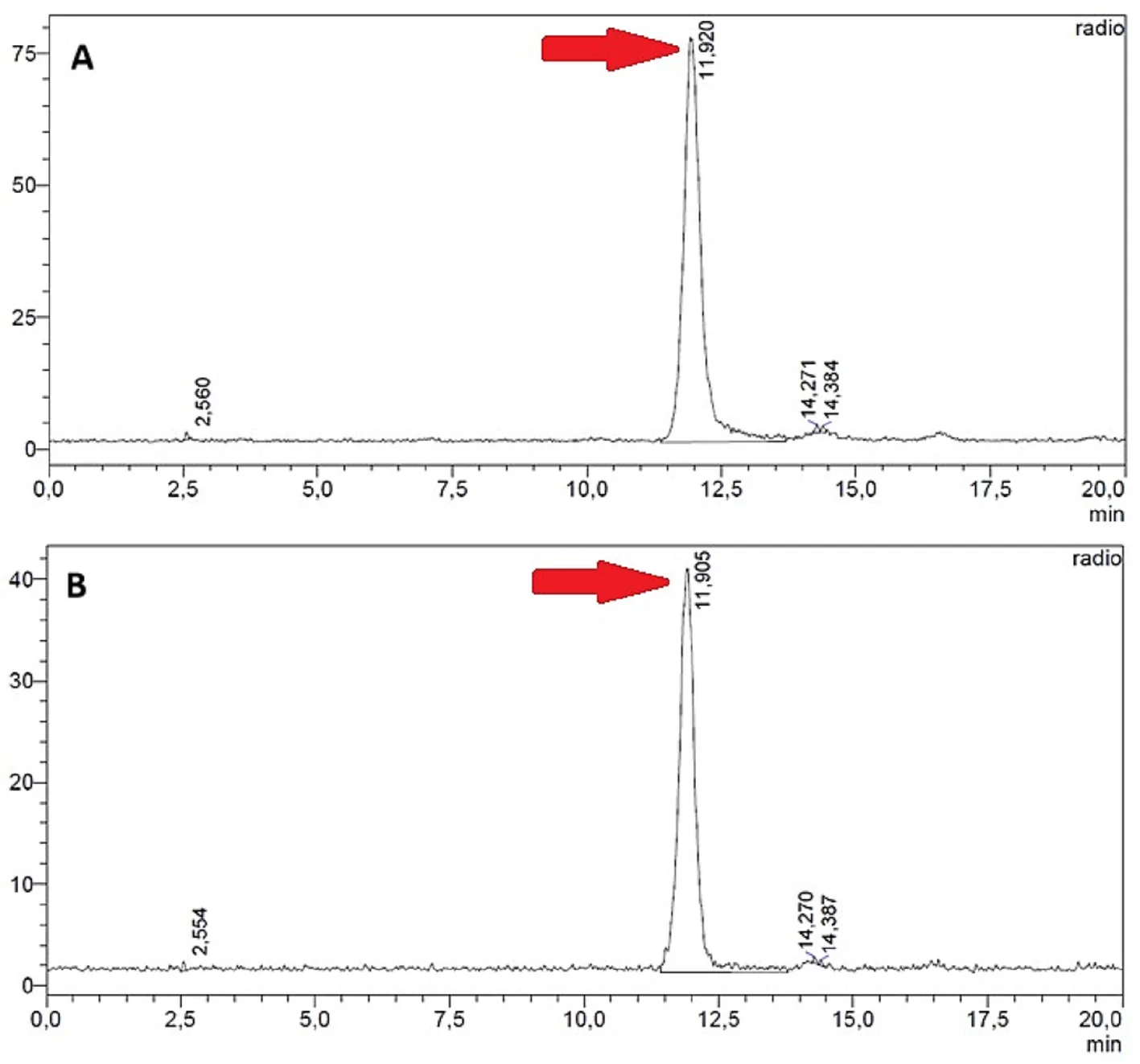
HPLC radiochromatogram of product 3: **a** immediately after preparation and **b** after 72 h of incubation in human serum at 37 °C

Radiolabelling procedures performed in this work were resulted with the formation of radiopharmaceutical agent for therapeutic use in human patients. For this reason, in vitro stability tests of products were performed by using isotonic saline and human serum, which were incubated at 25 °C and 37 °C during 72 h. Before performing in vitro tests, bacterial endotoxins levels were determined spectrophotometrically in all prepared products and in reference product. The level of endotoxins was found to be less than 5.0 EU / mL in all batches, which was within acceptable criteria defined by the European Pharmacopoeia monograph (European Pharmacopoeia 2025). Stability of the *in-house* prepared products in human serum was tested by HPLC method. It was assessed by monitoring the changes in peak profiles and retention time as showed in Fig. 6. Peak profiles of *in-house* prepared products and their retention times (11.9 min) after 72 h of incubation in human serum did not show any significant changes.

**Fig. 7:**
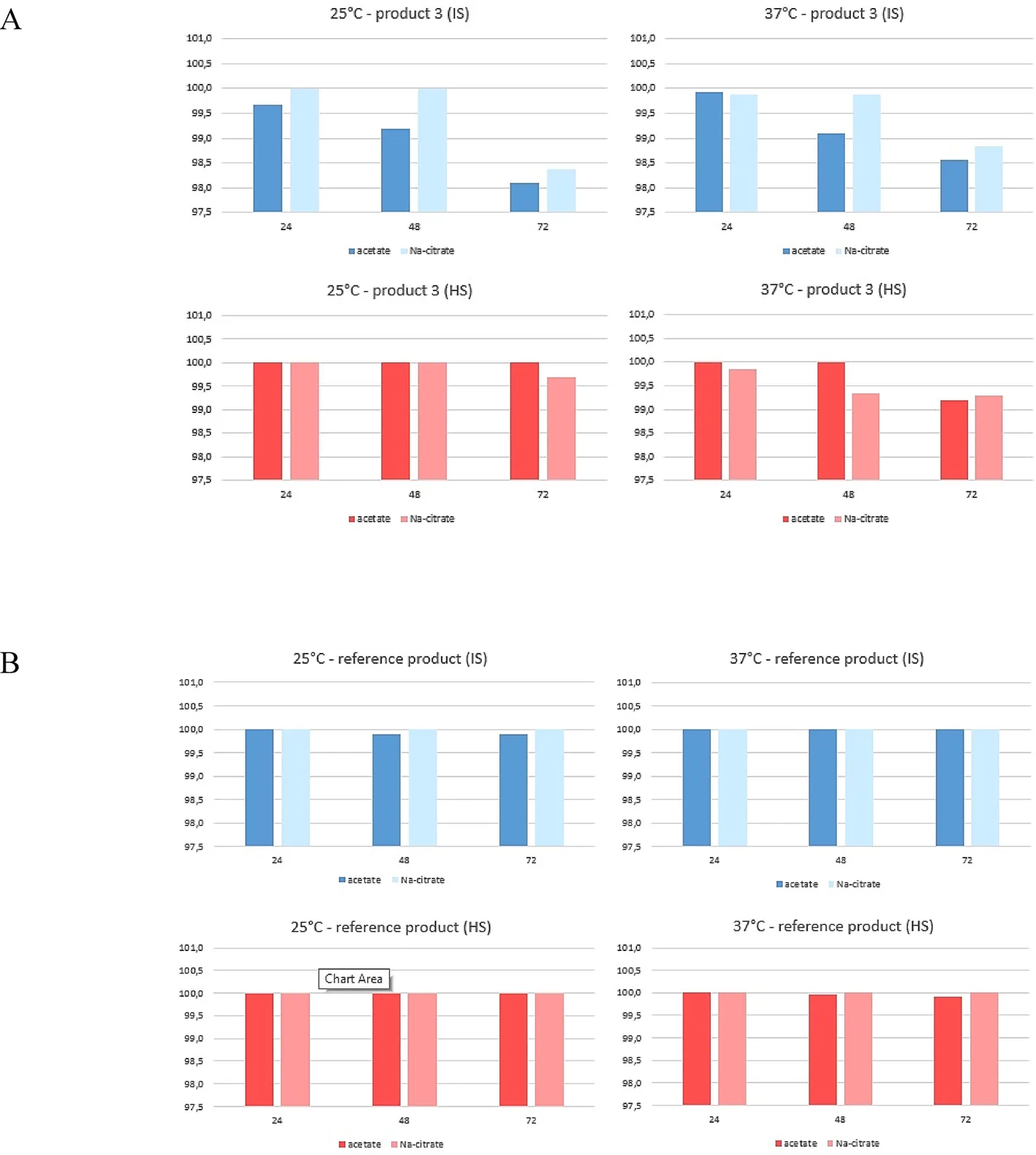
In vitro stability of [^177^Lu]Lu-PSMA-617 in isotonic solution (IS) and Human Serum (HS) at 25 °C and 37 °C during 72 h of incubation period (**a**) *in-house* prepared product, (**b**) reference product

Radiochemical purity of the radiopharmaceutical products used in experiments in vitro was obtained by TLC and HPLC measurements at 24 h, 48 h and 72 h after incubation in human serum and the results were presented in Fig. 7. It is showed that radiochemical purity of the products remained greater than 99% at 25 °C and also greater than 98% at 37 °C during 48 h after preparation of solutions. Undesired release of free ^177^Lu^3+^ was not observed during 24 h and 48 h of incubation period. The lowest observed difference regarding releasing free ^177^Lu^3+^ between 48 h and 72 h at 37 °C in human serum was 0.02. This is an important fact because free ^177^Lu^3+^ has shown to accumulate in the skeleton and in the liver, and it should be kept at minimum (Gillings et al. 2020). Slightly rise of concentrations of free ^177^Lu^3+^ (0.11–1.62%) was observed after 72 h from preparation. At this point, an increase of the ^177^Lu-colloid concentration (0.07–1.55%) in the observed samples which consisted of *in-house* prepared products was also detected.

The reference product Pluvicto^®^, which was used for comparison of properties, generally showed less changes in radiochemical purity which was tested in vitro during 72 h on similar manner (European Medicines Agency 2026). However, after 72 h from initial incubation, the release of free ^177^Lu^3+^ is observed in less extent (0.27–0.36%), than was noted in serum with *in-house* prepared products. The formation of the ^177^Lu-colloid also occurred with the use of reference product after 72 h (0.06–0.55%). The differences, which were noted between *in-house* products and reference products, might be explained by the mode of detection of the ^177^Lu on the strips during scanning with TLC scanner. All *in-house* products had lower initial activity of the ^177^Lu than the reference product. Therefore, significantly less counts were detected during creating the chromatograms, while peaks were broader. For that reason, the determination of the percentage of the complex for *in-house* batches is less precise than with the reference product of higher initial activity. However, the *in-house* prepared radiopharmaceutical showed satisfactory stability in vitro. The period of acceptable stability lasted 48 h, which is the period that is therapeutically recommended for administration of [^177^Lu]Lu-PSMA-617 to patients (Di Orio et al. 2022).

Human serum samples used for in vitro stability tests at 25 °C and 37 °C were left for 180 days and subsequently diluted and measured by ICP-MS method. The obtained results are presented in the Supplementary files (Table 1S). The concentration of formed stable isotopes of ^177^ Hf in solutions with *in-house* prepared products was significantly different than in referent product, which contained n.c.a. lutetium isotope. This indicates the support of the ICP-MS method in discriminating between the initial types of ca. or n.c.a ^177^Lu isotopes, which are used in the preparation of radiopharmaceuticals. The changes in temperature of incubation did not show significant concentration deviations of formed stable isotopes in periods of 24 and 48 h of incubation, which corresponds to the previously obtained results with HPLC method.

In general, when preparing radiopharmaceuticals by radiolabelling, it is desirable to maintain a minimum ratio of ligand to radionuclide. When formulation is based on carrier added ^177^Lu radionuclide, as was presented in described experiments, a ligand-to-metal ratio can be minimized with increase of the amount of radiometal in the reaction mixture. By using a fixed amount of 20 µg of the peptide PSMA, the variations of ligand-to-metal ratio were obtained by addition of 2.0 nmol to 10.2 nmol of ^177^LuCl_3_ solution. Satisfactory radiolabelling yield of 96% can be achieved already at a ligand-to-metal ratio ~ 2, as was obtained in batch 5. This example showed that an amount of 1.78 µg of ^177^Lu is incorporated into the peptide PSMA. Considering the specific activity of ^177^Lu, which amounted 370 GBq mg^− 1^ in starting LuCl_3_ solution, it can be counted that the amount of 1.78 µg of added metal could provide 658 MBq of activity in labelled product. Single patient dose which corresponds to activity of 7.4 GBq can be prepared by using minimal amount of 225 µg PSMA-617 ligand and 20 µg of ^177^Lu if the specific activity of ^177^Lu is 370 MBq mg^− 1^. In addition to the above, it is also confirmed that the addition of ethanol as a free radical scavenger during radiolabelling improves the radiochemical yield of prepared [^177^Lu]Lu-PSMA-617 agent.

## Discussion

The quality of radiopharmaceuticals must meet the acceptance criteria which are proposed in European Pharmacopoeia monograph (European Pharmacopoeia 2025). The published standards generally denote radiopharmaceutical [^177^Lu]Lu-PSMA-I&T, where DOTAGA chelator (1,4,7,10-tetraazacyclododecane-1-glutaric acid-,4,7,10-triacetic acid), a glutaric acid derivative of DOTA, is linked with peptide (Hartrampf et al. 2022; Hunt et al. 2026). Therefore, due to the related medical effects of both radiopharmaceuticals, the same quality testing approach was applied to the *in-house* product [^177^Lu]Lu-PSMA-617, which was obtained in the described experiments. The final products, which were obtained from nine variations of preparation parameters, were subdued to quality assessment procedures. This started with HPLC analysis of mixture of compounds ^nat^177Lu-PSMA-617 and PSMA-617 which showed a resolution of 4.22. This value implies exceptional separation of PSMA-617 peptide from lutetium complex. The resolution value is important for the expression of specificity since it must demonstrate that the radioactive product is resolved from any potentially interfering radioactive impurities. This is also defined by acceptance criteria of European Pharmacopoeia monograph, which recommends that specified resolution of these peaks in chromatograms needs to be greater than 2 (European Pharmacopoeia 2025; Gillings et al. 2020).

The radiochemical yields for all performed batches were calculated in accordance to previously published researches, i.e. by dividing the activity of the ^177^Lu at the end of the synthesis with the activity at the start of the synthesis without correction for radiochemical purity (Hooijman et al. 2022; Coenen et al. 2017). We showed that product of satisfactory radiochemical purity and yield could be obtained with relatively low consumption of starting reagents in automatic module.

To assess impurities in products obtained by *in-house* radiochemical labelling in all batches, HPLC and TLC methods were used. Both methods are recommended by the European Pharmacopoeia monographs for quality assessment of comparable radiopharmaceuticals (European Pharmacopoeia 2025; Gillings et al. 2020; Hooijman et al. 2022). The sensitive HPLC method is superior in detecting multiple types of possible impurities, while the alternative TLC method is characterized by its ease of application and speed of assessment (Hooijman et al. 2022). These features are also reflected in the results of the tests carried out, the results of which are showed in the Table 3. Free ^177^Lu^3+^ ions were determined by thin-layer chromatography and HPLC method. The results showed that using HPLC method, its presence was detected in all batches, unlike the TLC method. In the majority of results obtained from HPLC determination, the percentage was lower than recommended limit of free ^177^Lu^3+^ ions (≤ 3%) (Di Iorio et al. 2022). The exception is manifested in batch 7 where the radiolabelling was unsuccessful and all radionuclide content was eluted in free ion form. The acceptable limit for determination of the ^177^Lu-colloid with TLC method is < 2% according to monograph for similar compounds (European Pharmacopoeia 2025). Our experiments resulted with low content of colloidal impurities, which were generally very close to the method’s limit of quantification.

Amount of the peptide PSMA-617 after the performed labelling procedures showed a slight inconsistency compared to the initial mass of reagent used. The explanation for this discrepancy can be considered in the fact that the peak of the peptide in all batches was measured near the instrumental limit of detection. Also, this might be caused with relatively low activity of the ^177^LuCl_3_ starting solution, which was less than 1 GBq. The other probability of slightly higher measured values is broadening of the chromatogram peak of the peptide and therefore, all peptide peaks showed a bigger area after integration.

In addition to the determination of impurities from residual forms of ^177^Lu and peptides, tests of the influence of the addition of ethanol and Zn-DTPA on radiolabelled products were carried out. Generally, ethanol is used as a radical scavenger, while Zn-DTPA is used as a chelating agent (Larenkov et al. 2024; Wichmann et al. 2021; European Pharmacopoeia 2017). When compares batches 4 and 9 where ethanol was added in volume fraction of 7% and 10%, respectively, the better radiochemical purity was found in batch 9. The addition of 20 molar excess of Zn-DTPA ligand over PSMA-617, which was performed in batch 7 at the beginning of radiolabelling, resulted with no formation of active product. The absence of the product is caused by the complete transformation of ^177^Lu into a complex compound due to formation of ^177^Lu-DTPA (Watanabe et al. 2015). In batch 8, where Zn-DTPA was added at the end of the preparation in equimolar ratio, which was estimated to free ^177^Lu^3+^ concentration, the obtained product showed satisfactory radiochemical purity. In the reference product there was no evidence of any residual solvent such as ethanol. However, the slightly difference of measured radiochemical purity between the *in-house* products and the reference product may occur from slightly different pH values. The complex effects of pH on radiochemical purity and yield were thoroughly studied before (Larenkov et al. 2023). Generally, as pH increases, the degree of deprotonation of the chelating agent and the percentage of the deprotonated form increase, which affects the complex formation. Moreover, during the radiochemical labelling, the incorporation of ^177^Lu into the PSMA-617 might additionally influence on release of H^+^ ions (Larenkov et al. 2023, 2024). In our work, a slightly changes in pH values were examined. Relatively small volumes of the reagents were used in all performed experiments. Therefore, due to possible reagent losses it was not appropriate to check the pH of the reaction mixture before synthesis.

Radionuclidic stability in obtained products was examined by using HPGe detector using two energy peaks at 208 keV (^177^Lu) and at 228 keV (^177m^Lu). Energy peak at 113 keV (^177^Lu) was confirmed in the spectrum of the Pluvicto^®^ that was used as reference product and in spectra of all *in-house* prepared products. The missing ^177m^Lu energy peak points to the use of non-carrier added ^177^Lu isotope in production of reference sample. In contrast, radiolabelling procedures performed in this work rely on the use of carrier added ^177^Lu isotope in starting solution. The presence of ^177m^Lu was found to be less than 0.1%, which is lower than acceptable limits and in accordance to monograph of the European Pharmacopoeia for similar compounds (European Pharmacopoeia 2017). The radionuclidic purity of ^177^Lu-PSMA-617 remained unchanged over a 7-day period which ensures that no significant alterations in radionuclidic composition are expected within this time. The strong coordination chemistry of the DOTA chelator provided high kinetic and thermodynamic stability of the ^177^Lu complex which prevented radionuclide dissociation and the formation of radiometal-related impurities.

Stability tests of products obtained by applied procedures were also performed in isotonic saline and human serum samples. For whole set of samples during 24 h and 48 h of incubation period, the undesired release of free ^177^Lu^3+^ was not observed. The lowest observed difference regarding releasing free ^177^Lu^3+^ between 48 h and 72 h at 37 °C in human serum was 0.02. This is an important fact because free ^177^Lu^3+^ has shown to accumulate in the skeleton and in the liver, and therefore its concentration should be kept at minimum (Gillings et al. 2020). Slightly rise of concentrations of free ^177^Lu^3+^ (0.11–1.62%) was observed after 72 h from preparation. At this time, an increase of the ^177^Lu-colloid concentration (0.07–1.55%) in the observed samples which consisted of *in-house* prepared products was also detected. The reference product Pluvicto^®^, which was used for comparison of properties, and it was tested in vitro during 72 h on similar manner generally showed less changes in radiochemical purity (European Medicines Agency 2026). However, after 72 h from initial incubation, the release of free ^177^Lu^3+^ is observed in less extent (0.27–0.36%) than was noted in serum incubated with *in-house* prepared products. The formation of the ^177^Lu-colloid also occurred with the use of reference product after 72 h (0.06–0.55%). The differences, which were noted between *in-house* products and reference products, might be explained by the mode of detection of the ^177^Lu on the strips during scanning with TLC scanner. All *in-house* products had lower initial activity of the ^177^Lu than the reference product. Therefore, significantly less counts were detected during creating the chromatograms, while peaks were broader. For that reason, the determination of the percentage of the complex for *in-house* batches is less precise than with the reference product of higher initial activity. However, the *in-house* prepared radiopharmaceutical showed satisfactory stability in vitro. The period of acceptable stability lasted 48 h, which is the period that is therapeutically recommended for administration of [^177^Lu]Lu-PSMA-617 to patients (Di Orio et al. 2022).

It is already known that the direct production route of ^177^Lu radioisotope leads to formation of stable isotope ^177^Hf, which does not influence labelling conditions (Adya et al. 2022; Wallimann et al. 2024; Klika et al. 2024). The presence of metastable states ^177m^Lu at low concentration levels in the prepared products was noted by HPGe detection. Due to the longer half-lives of these states, additional testing of incubated serum samples was performed to estimate the proportion of hafnium isotopes formed. Therefore, the human serum samples, were analysed using ICP-MS method. This extremely sensitive method has been described somewhat less frequently in recent works related to the quality control of radiopharmaceuticals. Its advantages in the analysis of biological matrices go beyond the analytical capabilities of gamma spectrometry, however, it is generally most used for the examination of non-radioactive metal-peptide conjugates (Dash et al. 2015; Adya et al. 2022; Wallimann et al. 0.2024; Klika et al. 2024). For this reason, in this work, serum samples were analysed after a significant decrease in radioactivity of samples.

It is important to mention that some excipients (ethanol, peptide PSMA-617, ^177^LuCl_3_ solution, ethanol, Zn-DTPA) used for *in-house* radiolabelling were not of GMP grade. Nevertheless, implementation of fully GMP-compliant materials and microbiological testing would be recommendable for future scale-up and routine clinical production. In order to validate automatic module performances, for our further experiments, additional tests such as final product sterility and bioburden testing are also planned. Generally, by comparing the quality parameters of the *in-house* prepared product and the reference product, no significant deviations that could compromise the acceptability criteria set by the established standards were observed. Nonetheless, a product with a carrier added (ca.) isotope, such as was used in this work, is cost-effective due to lower production costs, which is also reflected in the price of the final product (Nanabala et al. 2022).

Furthermore, when preparing radiopharmaceuticals by radiolabelling, it is desirable to maintain a minimum ratio of ligand to radionuclide. When formulation is based on carrier added ^177^Lu radionuclide, as was presented in described experiments, a ligand-to-metal ratio can be minimized with increase of the amount of radiometal in the reaction mixture. By using a fixed amount of 20 µg of the peptide PSMA, the variations of ligand-to-metal ratio were obtained by addition of 2.0 nmol to 10.2 nmol of ^177^LuCl_3_ solution. Satisfactory radiolabelling yield of 96% can be achieved already at a ligand-to-metal ratio ~ 2, as was obtained in batch 5. This example showed that an amount of 1.78 µg of ^177^Lu is incorporated into the peptide PSMA. Considering the specific activity of ^177^Lu, which amounted 370 GBq mg^− 1^ in starting LuCl_3_ solution, it can be counted that the amount of 1.78 µg of added metal could provide 658 MBq of activity in labelled product. Single patient dose, which corresponds to activity of 7.4 GBq, can be prepared by using minimal amount of 225 µg PSMA-617 ligand and 20 µg of ^177^Lu if the specific activity of ^177^Lu is 370 MBq mg^− 1^. In addition to the above, it is also confirmed that the addition of ethanol as a free radical scavenger during radiolabelling improves the radiochemical yield of prepared [^177^Lu]Lu-PSMA-617 agent.

## Conclusions

In this work, [^177^Lu]Lu‑PSMA‑617 agent was successfully radiosynthetized using automated radiolabelling module. The variation of parameters such as ligand-to-metal ratio, pH of solution and addition of radical scavenger and complexing agent during radiolabelling were examined. The *in-house* preparation demonstrated the excellent radiochemical purity and high radiochemical yield of obtained products even with very low consumption of initial reagents. Stability studies in vitro confirmed that the radiopharmaceutical remained stable for up to 72 h in isotonic saline and in human serum under temperature of 25 °C and 37 °C. Synergistic application of inductively coupled plasma mass spectrometry (ICP-MS) and high-purity germanium (HPGe) gamma spectrometry enabled clear differentiation between no-carrier-added and carrier-added ^177^Lu isotope in prepared radiopharmaceutical. Quality tests, which were conducted according to the European Pharmacopoeia monograph for similar products, have shown the acceptability of the *in-house* prepared product for medical use. The main advantages of the described *in-house* radiolabelling procedure [^177^Lu]Lu-PSMA-617 are reduced preparation costs and, ultimately, better accessibility of ^177^Lu-radiopharmaceuticals.

## Supplementary Information

Below is the link to the electronic supplementary material.

Supplementary Material 1

## Data Availability

Not applicable.

## Abbreviations

^225^Ac: Actinium-225
^68^Ga: Gallium-68
c.a.: Carrier added
DOTA: 1,4,7,10-Tetraazacyclododecane-1,4,7,10-tetraacetic acid
DOTAGA: 1,4,7,10-Tetraazacyclododecane-1-glutaric acid-,4,7,10-triacetic acid
EOS: End of synthesis
GBq: Gigabecquerel
GMP: Good manufacturing practice
HCl: Hydrochloric acid
HPGe: High-purity germanium (detector)
HPLC: High performance liquid chromatography
ICP-MS: Inductively Coupled Plasma Mass Spectrometry
LAL: Limulus Amebocyte Lysate
LOQ: Limit of quantification
n.c.a.: Non-carrier added
PET: Positron emission tomography
Ph. Eur.: European Pharmacopoeia
PSMA: Prostate specific membrane antigen
RCP: Radiochemical purity
RCY: Radiochemical yield
TFA: Trifluoroacetic acid
TLC: Thin-layer chromatography
UV-Vis: Ultraviolet-Visible (detector)
Zn-DTPA: Pentetate zinc trisodium
*v/v*: Volume/volume
