# Supplementary material for: Development and evaluation of improved in-house procedure for radiolabelling of theranostic agent [177Lu]Lu-PSMA-617
Source: EJNMMI Radiopharm Chem. 2026 Jun 13;11:59. doi: 10.1186/s41181-026-00462-y (PMC13493687; doi:10.1186/s41181-026-00462-y)
Supplement: Supplementary file 1 [file 41181_2026_462_MOESM1_ESM.docx]

| Temperature |  | 25°C | 25°C | 25°C | 37°C | 37°C | 37°C |  | 25°C | 25°C | 25°C | 37°C | 37°C | 37°C |
| --- | --- | --- | --- | --- | --- | --- | --- | --- | --- | --- | --- | --- | --- | --- |
| Sample | **non**  **incubated** | **24 h** | **48 h** | **72 h** | **24 h** | **48 h** | **72 h** | **non**  **incubated** | **24 h** | **48 h** | **72 h** | **24 h** | **48 h** | **72 h** |
| µg L^-1^ | **^175^Lu** | **^175^Lu** | **^175^Lu** | **^175^Lu** | **^175^Lu** | **^175^Lu** | **^175^Lu** | **^177^ Hf** | **^177^ Hf** | **^177^ Hf** | **^177^ Hf** | **^177^ Hf** | **^177^ Hf** | **^177^ Hf** |
| product 1 | 10.607 | 5.889 | 6.481 | 6.112 | 6.584 | 6.184 | 6.579 | 58.617 | 32.335 | 35.783 | 32.776 | 36.315 | 33.211 | 36.131 |
| product 2 | 13.262 | 8.270 | 8.157 | 8.484 | 8.571 | 8.543 | 8.839 | 73.318 | 45.749 | 45.199 | 46.724 | 46.829 | 46.707 | 48.429 |
| product 3 | 17.609 | 11.855 | 11.880 | 11.107 | 11.890 | 11.698 | 13.086 | 96.434 | 64.412 | 64.267 | 61.166 | 64.365 | 64.931 | 71.791 |
| product 4 | 21.111 | 14.283 | 14.436 | 14.937 | 17.236 | 15.185 | 14.844 | 116.329 | 78.563 | 79.824 | 82.559 | 96.892 | 83.593 | 80.638 |
| product 5 | 32.083 | 21.693 | 25.646 | 24.394 | 21.912 | 22.543 | 23.292 | 249.119 | 167.467 | 198.704 | 187.353 | 168.347 | 172.395 | 181.142 |
| product 6 | 7.027 | 4.587 | 6.769 | N/A | 4.785 | 4.492 | 5.561 | 54.076 | 35.767 | 52.321 | N/A | 37.153 | 34.842 | 36.581 |
| product 7 | 18.613 | N/A | N/A | N/A | N/A | N/A | N/A | 143.560 | N/A | N/A | N/A | N/A | N/A | N/A |
| product 8 | 17.918 | 12.864 | 13.194 | 12.297 | 12.617 | 12.569 | 17.589 | 138.624 | 98.022 | 101.501 | 94.505 | 97.362 | 95.396 | 133.531 |
| product 9 | 14.633 | 8.794 | 8.518 | 7.229 | 9.380 | 10.522 | 9.317 | 113.383 | 68.637 | 65.234 | 54.214 | 70.730 | 79.111 | 71.581 |
| ref. product R | 68.987 | 26.452 | 27.482 | 28.260 | 26.808 | 26.766 | 28.498 | 0.906 | 0.523 | 0.366 | 0.302 | 0.220 | 0.242 | 0.223 |

Supplementary data:

Table 1S: Mass concentration of isotopes ^175^Lu and ^177^Hf determined by ICP-MS in products incubated in human serum at 25°C and	37°C during 72h

Figure 1S:

Picture of Gaia-Luna module set-up used for radiolabelling of [^177^Lu]Lu-PSMA-617
